# A randomised open-label cross-over study of inhaler errors, preference and time to achieve correct inhaler use in patients with COPD or asthma: comparison of ELLIPTA with other inhaler devices

**DOI:** 10.1038/npjpcrm.2016.79

**Published:** 2016-11-24

**Authors:** Job van der Palen, Mike Thomas, Henry Chrystyn, Raj K Sharma, Paul DLPM van der Valk, Martijn Goosens, Tom Wilkinson, Carol Stonham, Anoop J Chauhan, Varsha Imber, Chang-Qing Zhu, Henrik Svedsater, Neil C Barnes

**Affiliations:** 1 Department of Pulmonary Medicine, Medisch Spectrum Twente, Enschede, The Netherlands; 2 Department of Research Methodology, Measurement and Data Analysis, University of Twente, Enschede, The Netherlands; 3 Primary Care and Population Sciences, University of Southampton, Southampton, UK; 4 NIHR Southampton Respiratory Biomedical Research Unit, Southampton, UK; 5 Inhalation consultancy Ltd, Leeds, UK; 6 Respiratory Medical Franchise, GSK, Brentford, UK; 7 Department of Pulmonary Medicine, Gelre Ziekenhuizen, Zutphen, The Netherlands; 8 Clinical and Experimental Sciences, Faculty of Medicine, University of Southampton, Southampton, UK; 9 Minchinhampton Surgery, Stroud, UK; 10 Respiratory Department, Queen Alexandra Hospital, Portsmouth, UK; 11 MDC Global Clinical Development UK, Respiratory R&D, GSK, Uxbridge, UK; 12 Clinical Statistics, GSK, Uxbridge, UK; 13 Value Evidence Outcomes Respiratory, GSK, Brentford, UK

## Abstract

Errors in the use of different inhalers were investigated in patients naive to the devices under investigation in a multicentre, single-visit, randomised, open-label, cross-over study. Patients with chronic obstructive pulmonary disease (COPD) or asthma were assigned to ELLIPTA vs DISKUS (Accuhaler), metered-dose inhaler (MDI) or Turbuhaler. Patients with COPD were also assigned to ELLIPTA vs Handihaler or Breezhaler. Patients demonstrated inhaler use after reading the patient information leaflet (PIL). A trained investigator assessed critical errors (i.e., those likely to result in the inhalation of significantly reduced, minimal or no medication). If the patient made errors, the investigator demonstrated the correct use of the inhaler, and the patient demonstrated inhaler use again. Fewer COPD patients made critical errors with ELLIPTA after reading the PIL vs: DISKUS, 9/171 (5%) vs 75/171 (44%); MDI, 10/80 (13%) vs 48/80 (60%); Turbuhaler, 8/100 (8%) vs 44/100 (44%); Handihaler, 17/118 (14%) vs 57/118 (48%); Breezhaler, 13/98 (13%) vs 45/98 (46%; all *P*<0.001). Most patients (57–70%) made no errors using ELLIPTA and did not require investigator instruction. Instruction was required for DISKUS (65%), MDI (85%), Turbuhaler (71%), Handihaler (62%) and Breezhaler (56%). Fewer asthma patients made critical errors with ELLIPTA after reading the PIL vs: DISKUS (3/70 (4%) vs 9/70 (13%), *P*=0.221); MDI (2/32 (6%) vs 8/32 (25%), *P*=0.074) and significantly fewer vs Turbuhaler (3/60 (5%) vs 20/60 (33%), *P*<0.001). More asthma and COPD patients preferred ELLIPTA over the other devices (all *P*⩽0.002). Significantly, fewer COPD patients using ELLIPTA made critical errors after reading the PIL vs other inhalers. More asthma and COPD patients preferred ELLIPTA over comparator inhalers.

## Introduction

Inhaled medication has an important role in the treatment of chronic obstructive pulmonary disease (COPD) and asthma, and the effectiveness of inhaled medications is strongly influenced by the adherence to these medications.^1–5^ A decrease in medication delivery associated with an incorrect inhaler technique can lead to poor efficacy,^6,7^ with studies showing that >60% of patients use their inhaler ineffectively.^8–11^ When patients are prescribed an inhaled medicine, the choice of inhaler should, in part, be based on ease-of-use and on the ease with which a healthcare professional can teach correct technique.^12,13^ Correct inhaler technique involves some common steps for all devices although dose preparation and device orientation differ, highlighting the need for tailored patient training, testing and education.^14,15^ Training to minimise errors in the use of the device is essential to achieve the optimal drug effect, and all guidelines for asthma and COPD management recommend that a new inhaler device should not be prescribed until instruction from a health professional is provided and correct technique is demonstrated.^16,17^

Critical errors made when using inhalers, which significantly reduce or completely inhibit drug delivery, have been identified for various inhalation devices. Between 36 and 49% of patients with COPD perform critical inhaler errors with currently used inhalers,^18,19^ and in a review of studies by Cochrane *et al.*^20^ efficient inhalation technique was demonstrated by only 46–59% of asthma patients. The development of an easy-to-use inhaler device that delivers the drug to the lungs effectively is important.^21^ In addition, patient preference and satisfaction has been of increased interest over the past decade; preference for a particular medication or inhaler device may be associated with improved adherence with therapeutic regimens.^22^ The choice of inhalation device is an important consideration because it can influence patients’ adherence to treatment, and thus potentially affects the long-term outcome.^22^ Although device preference is interesting, errors in use are likely to have a greater effect on outcomes.

A dry powder inhaler, the ELLIPTA multi-dose inhaler, has been developed for the delivery of inhaled medication in patients with both COPD and asthma.^23^ This study aimed to assess the proportion of COPD and asthma patients making critical and overall errors when using the ELLIPTA inhaler and other commonly used commercially available inhaler devices such as the DISKUS/Accuhaler inhaler, metered-dose inhaler (MDI), Turbuhaler, Handihaler and Breezhaler. Critical errors were assessed by trained respiratory nurses after patients had read the patient information leaflet (PIL). The errors were assessed using an error checklist that was developed based on the respective PILs, existing literature and with the input from external experts. This study also assessed the time needed for instruction, ease-of-use and patient preference between the ELLIPTA inhaler and other commercially available inhaler devices.

## Results

### Baseline characteristics

#### COPD patients

A total of 567 patients with COPD were randomised, and all completed this single-visit study. The majority of patients were Caucasian (98%) and 74% had been diagnosed with COPD between 6 months and 10 years (Table 1). Seventy-five percent of the population had comorbid medical conditions in addition to COPD; the most commonly reported were hypertension (41%) and hypercholesterolaemia (26%). Few patients had rheumatoid arthritis or visual impairment (⩽10 patients within each substudy).

#### Asthma patients

A total of 162 patients with asthma were randomised, and all completed this single-visit study. The majority of patients (100 patients, 62%) had asthma for ⩾10 years, 68 patients (42%) had asthma for ⩾20 years and 35 patients (22%) had asthma for <5 years (Table 2). Only 32 of the planned 50 patients were entered into the ELLIPTA inhaler vs MDI substudy due to a lack of patients who were naive to the MDI. Less than half of the population (45%) had comorbid medical conditions in addition to asthma; the most commonly reported were hypertension (17%) and hypercholesterolaemia (14%). Rheumatoid arthritis was reported for 16 patients (10%) and visual impairment for only 1 patient.

### Critical errors (primary end point)

#### COPD patients

**Figure 1:**
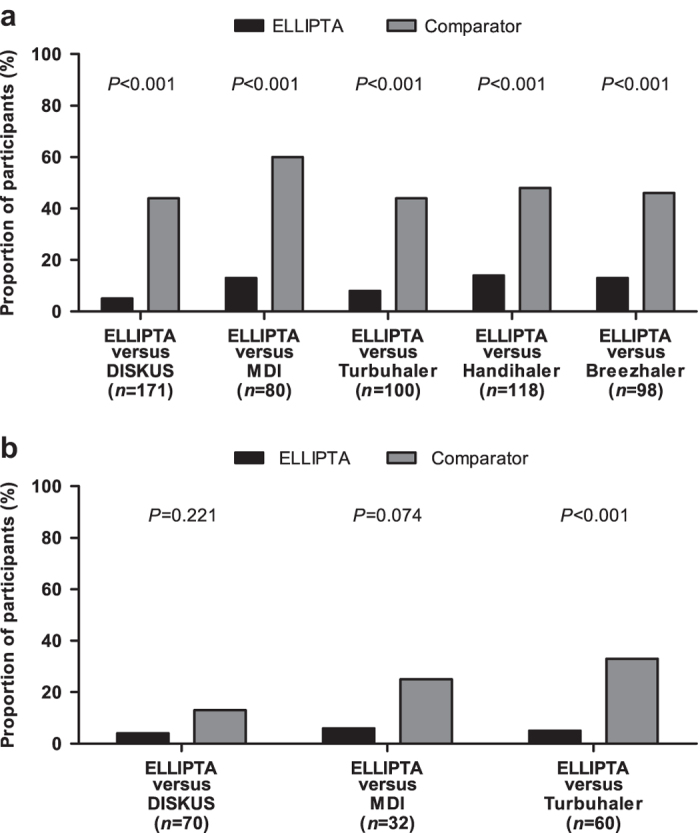
Percentage of patients with at least one critical error after reading the patient information leaflet in each substudy. (**a**) COPD patients and (**b**) asthma patients.

In all five substudies, after reading the PIL only, fewer patients had at least one critical error using the ELLIPTA inhaler compared with all five other inhalers (all *P*<0.001; Figure 1a; Table 3A). The most common critical error for the ELLIPTA inhaler was exhaling directly into the mouthpiece (31/567 patients, 5%); for DISKUS, the lever was not pushed back completely (56/171 patients, 33%); for MDI, it was poor press-and-breathe coordination (34/80 patients, 43%); for Turbuhaler, it was not twisting the base properly and hearing the click (29/100 patients, 29%); and for both Handihaler and Breezhaler, the capsule did not rattle (42/118 patients, 36% and 42/98 patients, 43%, respectively; Supplementary Table 2).

#### Asthma patients

In all three substudies, after reading the PIL only, fewer patients had at least one critical error using the ELLIPTA inhaler, albeit only statistically significant when compared with Turbuhaler, and borderline significant when compared with MDI (Figure 1b; Table 3B). The most common critical error for the ELLIPTA inhaler was exhaling directly into the mouthpiece (6/162 patients, 4%); for the DISKUS, it was the lever not being pushed back (9/70 patients, 13%); poor dose coordination (5/32 patients, 16%) was the most frequent critical error observed for the MDI; and not twisting the base properly and hearing the click (12/60 patients, 20%) for the Turbuhaler (Supplementary Table 3).

In the subgroup of 16 patients with concomitant rheumatoid arthritis across the three substudies, none made any critical errors with the ELLIPTA inhaler (*n*=16) or MDI (*n*=2), but two patients out of six (33%) made at least one critical error with DISKUS, and one patient out of eight (13%) made at least one critical error with Turbuhaler.

### Overall errors

#### COPD patients

In all five substudies, after reading the PIL only, fewer COPD patients had at least one overall error using the ELLIPTA inhaler compared with all five other inhalers (all *P*<0.001; Table 3A). The number of patients who had a non-critical error with the inhalation manoeuvre was 51/567 (9%) for the ELLIPTA inhaler, 35/171 (20%) for DISKUS, 25/80 (31%) for MDI, 18/100 (18%) for Turbuhaler, 13/118 (11%) for Handihaler and 20/98 (20%) for Breezhaler.

#### Asthma patients

In the three asthma substudies after reading the PIL, there was only a statistically significant difference between ELLIPTA and Turbuhaler in the number of patients with at least one overall error (Table 3B). The number of patients who had an error with the inhalation manoeuvre was 9 (6%) for the ELLIPTA inhaler, 6 (9%) for the DISKUS, 3 (9%) for the MDI and 18 (18%) for the Turbuhaler.

### Number of instructions from trained respiratory nurse

#### COPD patients

After reading the PIL, the majority of patients made no errors using the ELLIPTA inhaler (57–70% across the five substudies), thus not requiring instruction from the nurse. The majority of patients required nurse instruction for the other inhalers (65% DISKUS, 85% MDI, 71% Turbuhaler, 62% Handihaler and 56% Breezhaler).

In each substudy, fewer patients required more than one instruction for the ELLIPTA inhaler compared with DISKUS (7 vs 27 patients), MDI (3 vs 20 patients), Turbuhaler (6 vs 19 patients), Handihaler (13 vs 29 patients) and Breezhaler (2 vs 15 patients). The difference in the number of nurse instructions required until correct use was performed was statistically significant between the ELLIPTA inhaler and each of the other inhalers assessed (*P*<0.001).

#### Asthma patients

After reading the PIL, the majority of patients made no errors using the ELLIPTA inhaler (72%, 75% and 79% in substudies 1, 2 and 3, respectively) compared with DISKUS (69%), MDI (59%) and Turbuhaler (53%). Fewer patients required at least one study nurse instruction to perform correct inhaler use with the ELLIPTA inhaler compared with Turbuhaler (15/60 (25%) vs 28/60 (47%); *P*=0.004). The number of patients requiring study nurse instruction with ELLIPTA compared with DISKUS (15/70 (21%) vs 22/70 (31%)) and compared with MDI (9/32 (28%) vs 13/32 (41%)) was not statistically significantly different (*P*=0.174 and *P*=0.273, respectively).

The number of patients who required more than one instruction to demonstrate correct use was one for DISKUS, two for the ELLIPTA inhaler (one each in substudy 2 and 3), two for MDI and four for Turbuhaler. In substudy 3, more instructions were required for the correct use of the Turbuhaler compared with that of the ELLIPTA inhaler (*P*=0.004).

### Time to correct inhaler use

#### COPD patients

In all substudies, median T1 could only be determined for the ELLIPTA inhaler because for the other inhalers, more than half of the patients could not perform correct use after reading the PIL only. Time was censored for patients who did not use their inhaler correctly after three attempts. Median T3 was shorter for patients using the ELLIPTA inhaler compared with those using DISKUS (2.75 vs 3.93 min), MDI (3.79 vs 6.30 min), Turbuhaler (2.87 vs 7.80 min), Handihaler (4.32 vs 8.50 min) and Breezhaler (3.15 vs 8.44 min; all *P*<0.001).

#### Asthma patients

No statistically significant difference was found in the median T1 for patients using the ELLIPTA inhaler compared with those using the DISKUS (2.42 vs 3.13 min; *P*=0.46), MDI (2.98 vs 3.57 min; *P*=0.89) and Turbuhaler (4.25 vs 9.00 min; *P*=0.37).

No statistically significant difference was found in the median T3 for the ELLIPTA inhaler compared with that for the DISKUS (2.33 vs 2.86 min; *P*=0.89), MDI (2.82 vs 3.56 min; *P*=0.31) and Turbuhaler (4.17 vs 5.69 min; *P*=0.69).

### Ease-of-use and preference questionnaires

#### COPD patients

A larger proportion of patients in each substudy rated the ELLIPTA inhaler very easy or easy to use compared with DISKUS (97% vs 60%), MDI (92% vs 44%), Turbuhaler (96% vs 55%), Handihaler (98% vs 38%) or Breezhaler (94% vs 55%; all *P*<0.001). Larger proportions of patients responded that the ELLIPTA inhaler was very easy to use with regard to the dose counter, learning how to use the inhaler, handling the inhaler, preparing the inhaler for use and holding the inhaler while using it, compared with the proportions responding similarly for DISKUS, MDI, Turbuhaler, Handihaler and Breezhaler (Supplementary Table 4).

**Figure 2:**
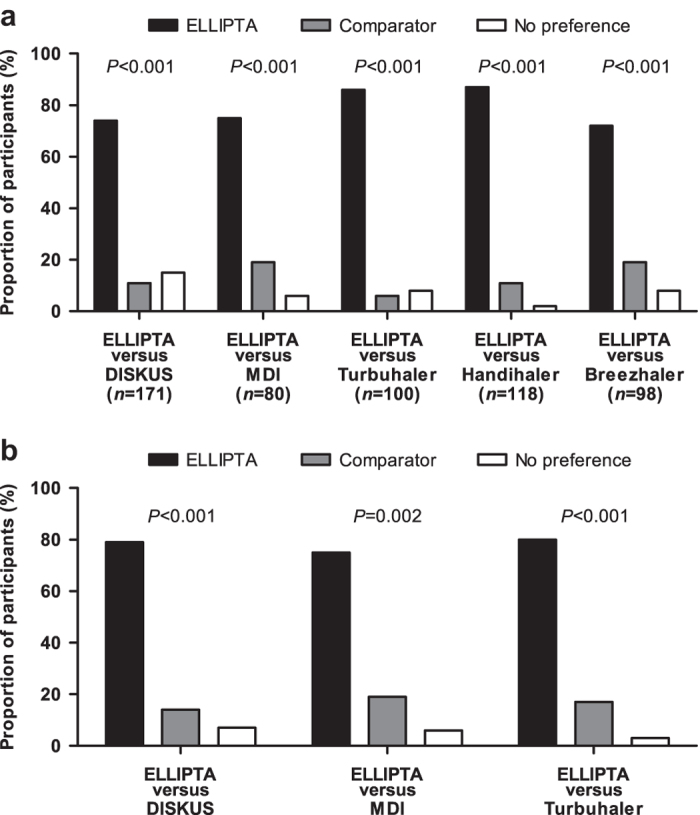
Overall device preference reported in each substudy. (**a**) COPD patients and (**b**) asthma patients.

Across the five substudies, patients preferred the ELLIPTA inhaler overall compared with the comparator devices (Figure 2). The majority of patients also preferred the ELLIPTA inhaler for most individual criteria (number of steps for correct use, time taken to use, size of the device, dose counter, comfort of mouthpiece and ease of opening; *P*<0.001) with some exceptions where there was no difference: for the size of the inhaler, similar proportions of patients preferred ELLIPTA inhaler and MDI (39 and 33%), ELLIPTA inhaler and Turbuhaler (44 and 38%), and ELLIPTA inhaler and Breezhaler (41 and 44%). For comfort of the mouthpiece, similar proportions of patients preferred ELLIPTA inhaler and Handihaler (33 and 31%), and ELLIPTA inhaler and Breezhaler (40 and 37%).

#### Asthma patients

The majority of patients in each substudy gave a statistically significant higher ease-of-use rating for the ELLIPTA inhaler compared with that for the other inhalers (*P*<0.001; Supplementary Table 5).

The majority of patients in each substudy preferred the ELLIPTA inhaler overall compared with the DISKUS (*P*<0.001), MDI (*P*=0.002) or Turbuhaler (*P*<0.001; Figure 2). Across the three substudies, the majority of patients preferred the ELLIPTA inhaler for all individual criteria compared with the other inhalers (number of steps for correct use, time taken to use, dose counter, comfort of mouthpiece and ease of opening; *P*<0.05; Supplementary Figure 1), except for the size of the inhaler.

### Safety

There were no adverse events reported throughout the study.

## Discussion

### Main findings

This study assessed how easy it was for patients who were naive to the inhaler devices under investigation to achieve good inhaler technique after reading the PIL. If good technique was not achieved after reading the PIL alone, patients received standardised instruction from a trained nurse. Technique was assessed by trained nurses using standardised error and critical error criteria, to attempt to reduce any bias. After reading the PIL alone, there were differences between devices in the proportion of patients achieving good technique. The majority of both COPD and asthma patients made no errors using the ELLIPTA inhaler and, therefore, did not require further instruction. However, a minority of patients did make errors, confirming that face-to-face instruction with a trained professional is required when commencing any new inhaler device. In contrast, using the comparator inhalers, the majority of COPD patients did make errors and thus required nurse instruction. COPD patients took less time to demonstrate correct inhaler use with the ELLIPTA inhaler after reading the PIL and receiving nurse instructions compared with the five comparator inhalers. For asthma patients, there was no significant difference in time to correct inhaler use between inhalers. The number of errors made by COPD and asthma patients across the substudies was consistent for the ELLIPTA inhaler.

### Interpretation of findings in relation to previously published work

Guidelines recommend that healthcare professionals should provide inhaler skills training,^24^ but in reality this may not always happen. Independently of the inhaler, a strong association has been found between inhaler misuse and lack of instruction by health caregivers.^6,25,26^ A lack of inhaler technique review was previously found to be significantly associated with making ⩾1 critical error.^3^ Our study aimed to assess critical errors, the teaching time required from a professional and preference for the inhalers.

The proportion of COPD patients making at least one critical error after reading the PIL was significantly lower with the ELLIPTA inhaler compared with all comparator inhalers. For asthma patients, the proportion making at least one critical error or overall error after reading the PIL was significantly lower with the ELLIPTA inhaler compared with the Turbuhaler with a nonsignificant trend for ELLIPTA compared with DISKUS and MDI. These findings are consistent with the previous data showing that error rates are higher for the MDI,^27^ Turbuhaler^6,28–30^ and Handihaler.^31^ The low error rates for the ELLIPTA inhaler compared with the other inhalers may reflect fewer steps, a shorter PIL and a more intuitive design.

Across all substudies, we observed good consistency in the error rates in both COPD and asthma patients using the ELLIPTA inhaler. The observed error rates for the comparator inhalers varied to some extent from those reported in the literature,^6,29,32–35^ possibly reflecting differences in the types of study conducted, the populations assessed and the assessment procedures used. Our study is likely to be more reproducible than those previously reported as patients in this study were recruited from routine care settings, were naive to the comparator inhalers, used standardised and explicit training and assessment protocols, and trained nursing assessors across the study sites. The observed critical error rates for devices other than the ELLIPTA inhaler were close to or lower than those assumed in the sample size calculation. The observed critical error rates for the ELLIPTA inhaler (5–14%) were lower than those assumed in the sample size calculation (20–30%). The standardised nurse training and the use of very specific checklists developed from the PILs may have also contributed to the low critical error rates. These errors rates are likely to be reproduced in future studies recruiting device-naive patients from ‘real-world’ clinical settings and to reflect the realities of errors associated with starting new inhaler devices in routine care.

A common error for the MDI is not to shake the inhaler before use. Although solution-formulated MDIs do not require shaking before firing, we know that many patients are switched between inhalers regularly. For these patients, it could be confusing if they were instructed differently for each MDI. Therefore, we assumed the ‘worst’ case scenario where shaking is necessary.

For one of the overall errors, the inhalation manoeuvre, it was expected that the number of errors would be similar for the dry powder inhaler devices, as in previous studies.^6,32^ However, fewer patients made an inhalation manoeuvre error with the ELLIPTA inhaler and the HandiHaler than other inhalers. This could be due to human factors such as preference and comfort of the mouthpiece as patients did favour the comfort of the mouthpiece of these two devices, or to the challenges of getting trained assessors to evaluate consistently. Recently, nurse assessments of the inhalation manoeuvre showed greater variability than those made by retrospective assessment of the videoed inhaler demonstrations.^36^

For COPD patients, the difference in the number of nurse instructions required was statistically significant between the ELLIPTA inhaler and each of the five comparator inhalers (*P*<0.001); the majority of patients required nurse instruction for the comparator inhalers.

Factors reported in the literature that have an impact on error frequency include older age^37^ and female gender.^38^ In addition, a higher frequency of errors has been found in patients with COPD,^39^ and a higher level of education has also been reported to be associated with a lower frequency of errors.^40^ In this study, the mean age of COPD patients enrolled was similar to that of the general COPD population. Literacy was assessed only in the UK populations, where between 94 and 96% of patients scored at the high school reading level. Therefore, literacy in this study may not be representative of a general real-world population, where health literacy/reading scores may be more variable.

Preference for an inhaler device may be associated with increased patient satisfaction and improved adherence to the treatment regimen.^22^ A larger proportion of COPD and asthma patients reported preference for the ELLIPTA inhaler compared with the comparator inhalers across most of the criteria in the preference questionnaire, except for the size of the inhaler and comfort of the mouthpiece, which was similar to some of the inhalers tested. Similar findings have been seen in previous studies.^41^

### Strengths and limitations of this study

Although all patients in this study were naive to the ELLIPTA inhaler and the comparator inhaler to remove prior experience influencing error rates, there are several limitations to the current research. Although every effort was made to standardise the training and training providers were attached to independent academic and clinical units with no contact with the study sponsor, the study was nevertheless open-label and involved subjective assessments and was hence open to potential nurse bias. There is also a tendency to over-assess in these studies.^36^ In addition, our study looked at initial training in a new device, and so does not provide information on the persistence of good technique and its relationship with long-term clinical outcomes. A study by Press *et al.*^42^ showed that patients’ technique wanes over time following nurse instruction and reading the PIL.

### Implications for future research, policy and practice

In summary, this study assessed the ease of mastery of technique for new inhaler devices in COPD and asthma patients recruited from the community and routine care settings, who were familiar with inhaler use but were naive to the devices assessed. The ELLIPTA inhaler was compared with other commonly used devices. Although some patients achieved good inhaler technique based on reading the manufacturers insert instructions only, instruction from a trained professional was needed for most patients. Poor inhaler technique is a common cause of treatment failure in patients with COPD and asthma.

### Conclusions and implications for future research, policy and practice

This study highlights the need for patients using a new device to be adequately trained and assessed at the time of initiation, for periodic reinforcement of training, and also the need to ensure that the device selected is suitable for the patient.

## Materials and methods

### Study design

These were randomised, open-label, placebo, cross-over, multicentre studies (GSK study numbers 200301 and 200330; ClinicalTrials.gov identifiers: NCT02184624 and NCT02195284). Patients were randomised at two centres in the United Kingdom and six in the Netherlands. Patients were required to attend one visit to the centre and could continue using their usual daily COPD or asthma medication during the study.

Patients with COPD were male or female, ⩾40 years of age and with a primary diagnosis of COPD as defined by the American Thoracic Society/European Respiratory Society.^43^ Patients with asthma were male or female, ⩾18 years of age, with a physician diagnosis of asthma and currently receiving treatment for asthma. Patients were required to be naive to ELLIPTA inhaler use and at least one other inhaler device (patients who were naive to the Breezhaler and Handihaler must have also been naive to all other inhaler devices that required a capsule). Those with a history of allergy/hypersensitivity to lactose/milk protein or magnesium stearate or to any other excipient found in commercially available inhaler devices were not enrolled in this study. COPD patients with a current diagnosis of asthma were excluded from the COPD study arms; asthma patients with a current diagnosis of COPD were excluded from the asthma study arms.

Patients received inactive treatment (placebo) via the ELLIPTA inhaler and were assigned to one of the other comparator inhaler devices to which they were naive. In substudy 1, COPD and asthma patients used the ELLIPTA inhaler and DISKUS/Accuhaler inhaler; in substudy 2, they used ELLIPTA inhaler and MDI; and in substudy 3, they used ELLIPTA inhaler and Turbuhaler. In substudy 4, COPD patients only used the ELLIPTA inhaler and Handihaler, and in substudy 5, COPD patients only used the ELLIPTA inhaler and Breezhaler (Supplementary Table 1). The sequence of using the two inhaler devices within each substudy was randomised.

The study was conducted in accordance with the International Conference on Harmonization Good Clinical Practice guidelines (ICH-GCP) and the 2008 version of the Declaration of Helsinki. All patients provided written informed consent before participation. The ethics and review boards of participating institutions approved the protocol before commencement of the study (in the Netherlands, the protocol was approved by the Medical Ethical Committee Twente; this approval was valid for all study sites in the Netherlands; in the United Kingdom, the protocol was approved by the West of Scotland Research Ethics Committee 4 and was valid for the UK sites).

### Outcomes and assessments

#### Assessment of errors

Respiratory nurses were trained in the correct inhaler use of all inhalers before the study by face-to-face and videotaped instruction training based on the checklist of errors (Table 4). After randomisation, patients were asked to read the PIL of the first device and then were asked to perform inhaler use. Overall errors made by the patient while using the first inhaler were recorded by a trained respiratory nurse. If the patient made any errors in inhaler use after reading the PIL, the nurse demonstrated the correct use of the inhaler to the patient, and the patient was asked to perform the inhaler use again. If the patient continued to make errors in inhaler use, the nurse demonstrated the process again up to a maximum of three times. After completing the process with the first inhaler, the same procedures were followed for the second inhaler.

### Time taken to correctly use the device

The trained respiratory nurse recorded time taken until correct use of the device. T1 was the time from when the patient started to read the PIL until using the inhaler (with no nurse support). T2 was the time from when the nurse started to demonstrate/instruct device use until up to three given instructions and demonstration of inhaler use. T3 was the sum of T1+T2 giving the time from when the patient started to read the PIL until up to three instructions and demonstration of inhaler use. Where no nurse instruction was required, T2 was equal to zero.

### Number of instructions from trained respiratory nurses

The number of instructions (maximum three times) from the nurse that were needed to demonstrate correct inhalation technique was recorded.

### Assessments of preference and ease-of-use

After completing the demonstration procedures of the two inhalers, the nurse asked the participant questions using two questionnaires; the ease-of-use questionnaire followed by the preference questionnaire.

### Safety

Safety was assessed by monitoring adverse events.

### Statistical analysis

The sample size calculation for each substudy was based on the results from a pilot study that examined error rates for ELLIPTA inhaler and those from other published device critical error studies for the relevant inhaler devices. For COPD patients, the critical error rate for the ELLIPTA inhaler, DISKUS inhaler, Handihaler, Turbuhaler, Breezhaler and MDI was assumed to be 30%, 50%, 55%, 58%, 58% and 62%, respectively.^6,27–30,32–34,44^ The rate for patients making at least one critical error for both inhaler devices in each substudy was assumed to be 30% of the ELLIPTA inhaler error rate, i.e., 9%.

For asthma patients, the critical error rate for the ELLIPTA inhaler, DISKUS, Turbuhaler and MDI was assumed to be 20%, 50%, 58% and 62%, respectively.^29,32–35^ The rate for patients making at least one critical error for both inhaler devices in each substudy was assumed to be 30% of the ELLIPTA inhaler error rate, i.e., 6%.

A patient who had a critical error with both devices or who had no critical errors with both devices did not provide any information about the superiority of either device. Only those patients who had critical error(s) in one device but not the other device were counted in the comparison. A McNemar test with a two-sided 5% significance level was used in the calculations, assuming that there were no period effects.

A total of 570 COPD patients (170, 120, 100, 100 and 80 patients for the ELLIPTA inhaler vs: DISKUS inhaler, Handihaler, Turbuhaler, Breezhaler and MDI, respectively) provided at least 90% power to detect a difference in the proportion of patients who made at least one critical error after reading the PIL comparing the ELLIPTA inhaler with each comparator inhaler device. A total of 180 asthma patients (70, 60 and 50 patients for the ELLIPTA vs: DISKUS/Accuhaler, Turbuhaler and MDI, respectively) provided at least 90% power to detect a difference in the proportion of patients who made at least one critical error after reading the PIL comparing the ELLIPTA inhaler with each comparator inhaler device.

The primary end point (proportion of patients having at least one critical error using the inhaler after reading the PIL) was analysed using a Cochran–Mantel–Haenszel test adjusted for country. A critical error was defined as an error that was likely to result in no, or minimal (i.e., significantly reduced) medication being inhaled. Overall errors were defined as any errors including critical errors. The proportion of patients having at least one overall error after reading the PIL was analysed in the same manner as the primary end point. The difference in the number of nurse instructions required for correct inhaler use between the two inhalers was analysed using a Wilcoxon signed-rank test. Patient ease-of-use rating and inhaler preference were analysed using a Cochran–Mantel–Haenszel test adjusted for country. Median times to correctly using inhaler were estimated using Kaplan–Meier analysis, where time was censored for patients who did not use the inhaler correctly. Log-rank analysis was used in a *post hoc* analysis comparing time to correctly completing inhaler use following nurse instruction.

## Figures and Tables

**Table 1:** Demographic characteristics of the COPD population

*Demographic characteristic*	*ELLIPTA vs*					
	*Substudy 1* *DISKUS* *(*N*=171)*	*Substudy 2* *MDI* *(*N*=80)*	*Substudy 3* *Turbuhaler* *(*N*=100)*	*Substudy 4* *Handihaler* *(*N*=118)*	*Substudy 5* *Breezhaler (*N*=98)*	*Total (*N*=567)*
*Age (years)*						
Mean	67.8	65.6	68.1	67.3	67.2	67.3
(s.d.)	7.4	8.8	8.7	9.0	8.2	8.3
						
*Sex,*n* (%)*						
Male	103 (60)	53 (66)	67 (67)	72 (61)	47 (48)	342 (60)
						
*Body mass index (kg/m*^*2*^)						
Mean	28.0	27.3	28.5	27.5	28.4	28.0
(s.d.)	6.4	4.6	5.1	5.5	6.7	5.8
						
*COPD history,*n* (%)*						
<6 months to <10 years	135 (79)	59 (74)	62 (62)	92 (78)	74 (76)	422 (74)
⩾10 to <25 years	29 (17)	18 (22)	32 (32)	23 (19)	21 (21)	123 (22)
⩾25 years	7 (4)	3 (4)	6 (6)	3 (3)	3 (3)	22 (4)

Abbreviations: COPD, chronic obstructive pulmonary disease; MDI, metered-dose inhaler.

**Table 2:** Demographic characteristics of the asthma population

*Demographic characteristic*	*ELLIPTA vs*			
	*Substudy 1* *DISKUS* *(*N*=70)*	*Substudy 2* *MDI (*N*=32)*	*Substudy 3* *Turbuhaler* *(*N*=60)*	*Total* *(*N*=162)*
*Age (years)*				
Mean (s.d.)	48.6 (17.8)	41.6 (16.4)	46.1 (18.5)	48.6 (17.8)
				
*Sex,*n* (%)*				
Male	33 (47)	15 (47)	20 (33)	68 (42)
				
*Race,*n *(%)*				
White	69 (99)	32 (100)	59 (98)	160 (99)
Asian	1 (<1)	0	0	1 (<1)
				
*Body mass index (kg/m*^*2*^)				
Mean	27.2	27.5	27.9	27.5
(s.d.)	5.1	5.7	6.1	5.6
				
*Asthma history,*n* (%)*				
<1 year	0	2 (6)	1 (2)	3 (1)
⩾1 to <5 years	11 (16)	6 (19)	15 (25)	32 (20)
⩾5 to <15 years	19 (27)	14 (44)	15 (25)	48 (30)
⩾15 years	40 (57)	10 (32)	29 (48)	79 (49)

Abbreviation: MDI, metered-dose inhaler.

**Table 3:** Summary of number of patients with at least one critical error or overall error using ELLIPTA vs comparator inhaler

*(A) COPD patients*					
	*ELLIPTA vs*				
	*DISKUS* *(*N*=171)*	*MDI* *(*N*=80)*	*Turbuhaler* *(*N*=100)*	*Handihaler* *(*N*=118)*	*Breezhaler* *(*N*=98)*
Critical errors, *n* (%)	9 (5) vs 75 (44)	10 (13) vs 48 (60)	8 (8) vs 44 (44)	17 (14) vs 57 (48)	13 (13) vs 45 (46)
*P* value	<0.001	<0.001	<0.001	<0.001	<0.001
Overall errors, *n* (%)	52 (30) vs 112 (65)	25 (31) vs 68 (85)	31 (31) vs 71 (71)	51 (43) vs 73 (62)	30 (31) vs 55 (56)
*P* value	<0.001	<0.001	<0.001	<0.001	<0.001

*(B) Asthma patients*			
	*ELLIPTA vs*		
	*DISKUS* *(*N*=70)*	*MDI* *(*N*=32)*	*Turbuhaler* *(*N*=60)*
Critical errors, n (%)	3 (4) vs 9 (13)	2 (6) vs 8 (25)	3 (5) vs 20 (33)
*P*value	0.221	0.074	<0.001
Overall errors, *n* (%)	15 (21) vs 22 (31)	9 (28) vs 13 (41)	15 (25) vs 28 (47)
*P* value	0.186	0.217	0.022

Abbreviation: COPD, chronic obstructive pulmonary disease.

**Table 4:**
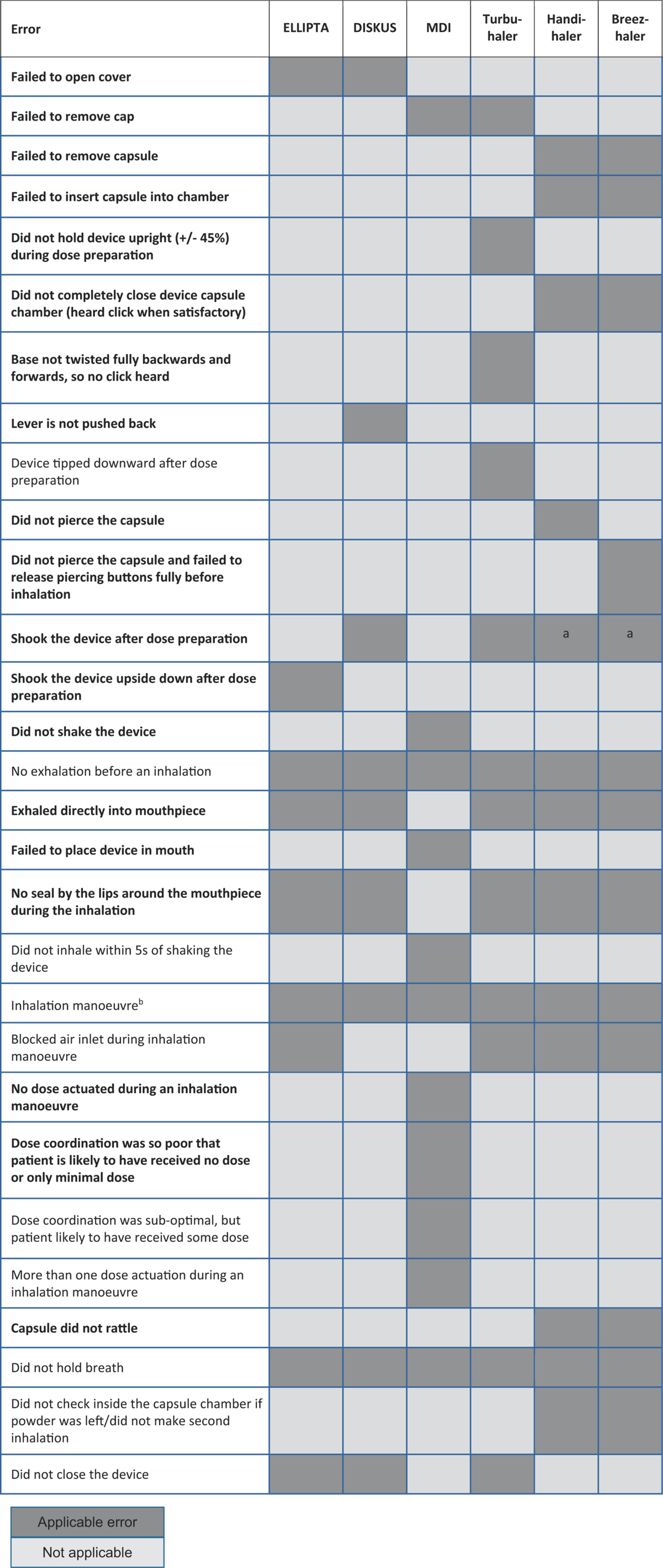
Errors checklist (critical errors in bold)
