# A randomised open-label cross-over study of inhaler errors, preference and time to achieve correct inhaler use in patients with COPD or asthma: comparison of ELLIPTA with other inhaler devices

**DOI:** 10.1038/npjpcrm.2017.1

**Published:** 2017-03-23

**Authors:** Job van der Palen, Mike Thomas, Henry Chrystyn, Raj K Sharma, Paul DLPM van der Valk, Martijn Goosens, Tom Wilkinson, Carol Stonham, Anoop J Chauhan, Varsha Imber, Chang-Qing Zhu, Henrik Svedsater, Neil C Barnes

**Correction to:** *npj Primary Care Respiratory Medicine* (2016) **26**, 16079; doi:10.1038/npjpcrm.2016.79; published online 24 November 2016

In the original version of the article, there were typographical errors that have been corrected in the html and pdf versions of the article. On page 2, column 1, under Results, ‘Caucasian (98%)’ was replaced with ‘Caucasian (>99%)’; in Table 2, mean age in total population ‘48.6 (17.8)’ was replaced with ‘46.3 (17.9)’; and in Table 4, ‘Did not hold device upright (±45%) during dose preparation’ was replaced with ‘Did not hold device upright (±45°) during dose preparation’. On page 4, column 1, under ‘COPD patients’, the text ‘all *P*<0.001’ was deleted.

