# Supplementary material for: A randomised open-label cross-over study of inhaler errors, preference and time to achieve correct inhaler use in patients with COPD or asthma: comparison of ELLIPTA with other inhaler devices
Source: NPJ Prim Care Respir Med. 2016 Nov 24;26:16079–. doi: 10.1038/npjpcrm.2016.79 (PMC5122307; doi:10.1038/npjpcrm.2016.79)
Supplement: Supplementary Information [file npjpcrm201679-s1.doc]

**Supplementary Figure 1**. Inhaler device preference ELLIPTA**®** (A) versus DISKUS; (B) versus MDI and (C) versus Turbuhaler, as reported by asthma patients

**
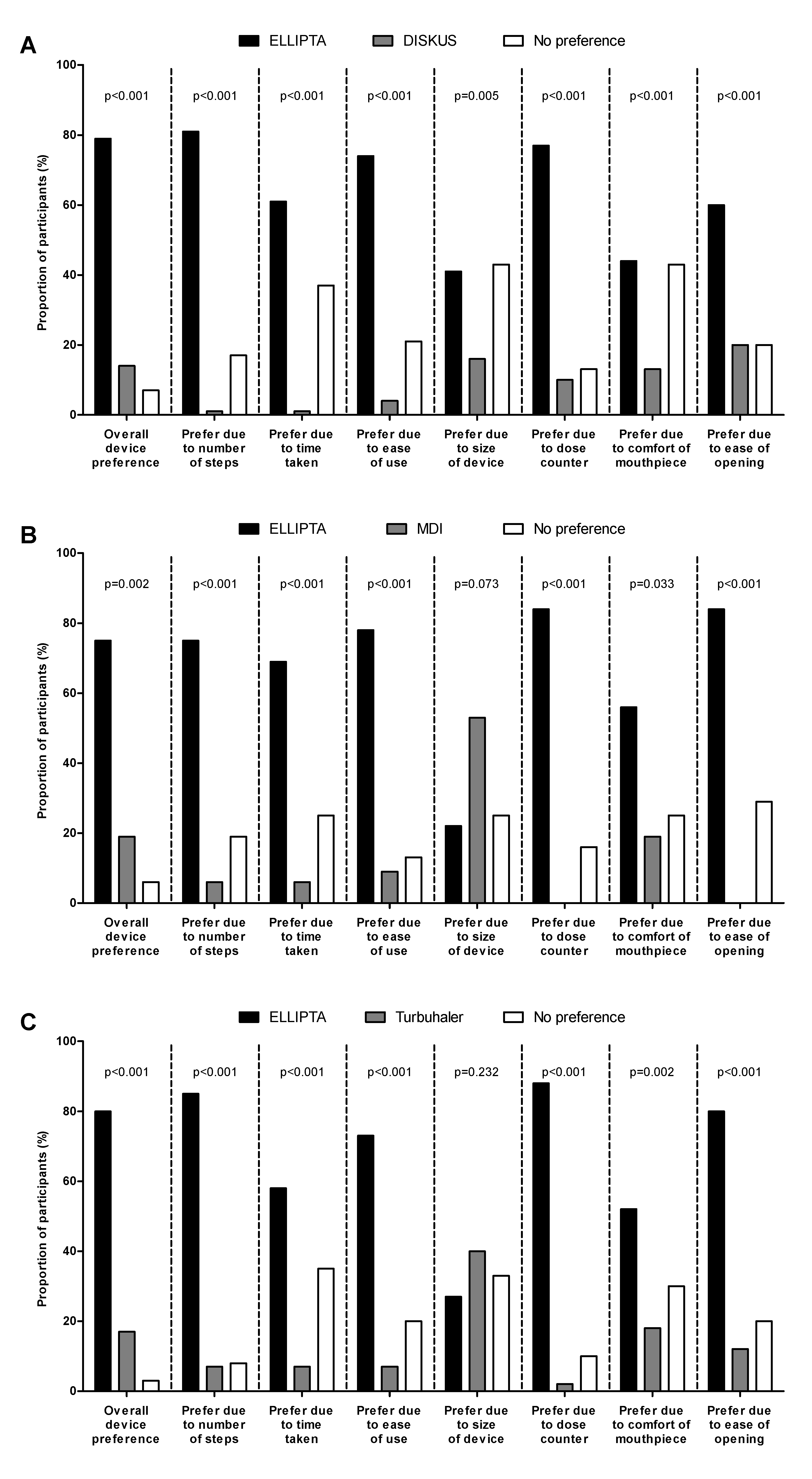
**

**Supplementary Table 1:** Summary of substudies in COPD and asthma patients

|  | **COPD patients** | **Asthma patients** |
| --- | --- | --- |
| **Substudy 1** | ELLIPTA first and then DISKUS/Accuhaler  OR  DISKUS/Accuhaler first and then ELLIPTA | ELLIPTA first and then DISKUS/Accuhaler  OR  DISKUS/Accuhaler first and then ELLIPTA |
| **Substudy 2** | ELLIPTA first and then MDI  OR  MDI first and then ELLIPTA | ELLIPTA first and then MDI  OR  MDI first and then ELLIPTA |
| **Substudy 3** | ELLIPTA first and then Turbuhaler  OR  Turbuhaler first and then ELLIPTA | ELLIPTA first and then Turbuhaler  OR  Turbuhaler first and then ELLIPTA |
| **Substudy 4** | ELLIPTA first and then Handihaler  OR  Handihaler first and then ELLIPTA |  |
| **Substudy 4** | ELLIPTA first and then Breezhaler  OR  Breezhaler first and then ELLIPTA |  |

**Supplementary Table 2**: Number of COPD patients who demonstrated critical errors in each substudy

| **Error** | **ELLIPTA**  **N=567** | **DISKUS**  **(N=171)** | **Turbuhaler**  **(N=100)** | **MDI**  **(N=80)** | **Breezhaler**  **(N=118)** | **Handihaler**  **(N=98)** |
| --- | --- | --- | --- | --- | --- | --- |
| Failed to open cover | n=9 | n=2 |  |  |  |  |
| Failed to remove cap |  |  | n=4 | n=5 |  |  |
| Failed to remove capsule |  |  |  |  | n=1 | n=8 |
| Lever is not pushed back |  | n=56 |  |  |  |  |
| Did not hold device upright (±45% OK) during dose preparation |  |  | n=14 |  |  |  |
| Base not twisted fully backwards and forwards, no |  |  | n=29 |  |  |  |
| Failed to insert capsule into the chamber |  |  |  |  | n=4 | n=8 |
| Did not completely close device capsule chamber |  |  |  |  | n=3 | n=7 |
| Did not pierce the capsule |  |  |  |  | n=14 | n=27 |
| Shook the device after dose preparation |  | n=8 | n=1 |  |  |  |
| Did not shake the device |  |  |  | n=23 |  |  |
| Failed to place device in mouth |  |  |  | n=1 |  |  |
| Shook the device upside down after dose preparation | n=7 |  |  |  |  |  |
| Exhaled directly into mouthpiece | n=31 | n=16 | n=7 |  | n=6 | n=6 |
| No seal by the lips round the mouthpiece during the inhalation | n=17 | n=13 | n=5 |  | n=4 | n=4 |
| No dose actuated during an inhalation manoeuvre |  |  |  | n=21 |  |  |
| Dose coordination was so poor that patient is likely to have received no dose or only received minimal dose |  |  |  | n=34 |  |  |
| Capsule did not rattle |  |  |  |  | n=42 | n=42 |

**Supplementary Table 3**: Number of asthma patients who demonstrated critical errors in each substudy

| **Error** | **ELLIPTA**  **N=162** | **DISKUS**  **N=70** | **Turbuhaler**  **N=60** | **MDI**  **N=32** |
| --- | --- | --- | --- | --- |
| Failed to open cover | 0 | n=1 |  |  |
| Failed to remove cap |  |  | n=1 | 0 |
| Lever is not pushed back |  | n=9 |  |  |
| Did not hold device upright (±45% OK) during dose preparation |  |  | n=4 |  |
| Base not twisted fully backwards and forwards, so no click heard |  |  | n=12 |  |
| Shook the device after dose preparation |  | 0 | n=1 |  |
| Did not shake the device |  |  |  | n=4 |
| Failed to place device in mouth |  |  |  | 0 |
| Shook the device upside down after dose preparation | n=3 |  |  |  |
| Exhaled directly into mouthpiece | n=6 | 0 | n=6 |  |
| No seal by the lips round the mouthpiece during the inhalation | 0 | 0 | n=2 |  |
| No dose actuated during an inhalation manoeuvre |  |  |  | n=1 |
| Dose coordination was so poor that patient is likely to have received no dose or only received minimal dose |  |  |  | n=5 |
|  | | | | |

**Supplementary Table 4:** Summary of ease of use questionnaire for each inhaler device; number (%) of COPD patients who responded to each question

|  |  | Sub study 1  (N=171) | | Sub study 2  (N=80) | | Sub study 3  (N=100) | | Sub study 4  (N=118) | | Sub study 5  (N=98) | |
| --- | --- | --- | --- | --- | --- | --- | --- | --- | --- | --- | --- |
|  |  | ELLIPTA | DISKUS | ELLIPTA | MDI | ELLIPTA | Turbuhaler | ELLIPTA | Handihaler | ELLIPTA | Breezhaler |
| How do you rate ease of use of the inhaler? | Very easy/easy  Neutral  Difficult  Very difficult | 165 (97)  6 (4)  0  0 | 104 (60)  40 (23)  26 (15)  1 (<1) | 73 (92)  5 (6)  1 (1)  1 (1) | 35 (44)  23 (29)  17 (21)  5 (6) | 96 (96)  4 (4)  0  0 | 55 (55)  22 (22)  20 (20)  3 (3) | 115 (8)  3 (92)  0  0 | 45 (38)  43 (36)  25 (21)  5 (4) | 92 (94)  3 (3)  3 (3)  0 | 54  30 (31)  12 (12)  2 (2) |
| How easily are you able to tell how many doses of medication are left in the inhaler? | Very easy/easy  Neutral  Difficult  Very difficult  Missing | 170 (99)  1 (<1)  0  0  0 | 105 (61)  37 (22)  26 (15)  3 (2)  0 | 77 (96)  2 (3)  1 (1)  0  0 | 51 (63)  14 (18)  10 (13)  5 (6)  0 | 100 (100)  0  0  0  0 | 45 (45)  19 (19)  30 (30)  6 (6)  0 | 115 (97)  2 (2)  1 (<1)  0  0 | 50(42)  50 (42)  15 (13)  1 (<1)  2 (2) | 97 (99)  1 (1)  0  0  0 | 60  21 (21)  13 (13)  1 91)  3 |
| How do you rate the ease of learning how to use the inhaler? | Very easy/easy  Neutral  Difficult  Very difficult | 163 (95)  8 (5)  0  0 | 109 (64)  43 (25)  19 (11)  0 | 74 (92)  3 (4)  2 (3)  1 (1) | 48 (60)  16 (20)  11 (14)  5 (6) | 94 (94)  5 (5)  0  0 | 53 (53)  21 (21)  23 (23)  3 (3) | 111 (94)  6 (5)  1 (<1)  0 | 72 (61)  25 (21)  18 (15)  3 (3) | 95 (97)  2 (2)  1 (1)  0 | 68 (70)  18 (18)  10 (10)  2 (2) |
| How do you rate the ease of handling the inhaler? | Very easy/easy  Neutral  Difficult  Very difficult | 165 (97)  5 (3)  1(<1)  0 | 120 (70)  30 (18)  20 (12)  1 (<1) | 76 (95)  2 (3)  1 (1)  1 (1) | 47 (58)  15 (19)  15 (19)  3 (4%) | 94 (94)  5 (5)  1 (1)  0 | 60 (60)  22 (22)  15 (15)  3 (3) | 112 (95)  5 (4)  1 (<1)  0 | 67  28 (24)  21 (18)  2 (2) | 91 (93)  5 (5)  2 (2)  0 | 67 (69)  21 (21)  10 (10)  0 |
| How do you rate the ease of preparing the inhaler for use? | Very easy/easy  Neutral  Difficult  Very difficult | 167 (98)  3 (2)  1 (<1)  0 | 124 (73)  31 (18)  14 (8)  2 (1) | 78 (97)  2 (3)  0  0 | 56 (70)  14 (18)  9 (11)  1 (1) | 96 (96)  3 (3)  1 (1)  0 | 71 (71)  15 (15)  10 (10)  4 (4) | 114 (96)  4 (3)  0  0 | 57 (48)  30 (25)  28 (24)  3 (3) | 95 (97)  2 (2)  1 (1)  0 | 55 (56)  29 (30)  12 (12)  2 (2) |
| How do you rate the ease of holding the inhaler while using it? | Very easy/easy  Neutral  Difficult  Very difficult  Missing | 165 (96)  3 (2)  2 (1)  0  1 (<1%) | 122 (71)  33 (19)  13 (8)  2 (1%)  1 (<1%) | 74 (92)  5 (6)  0  1 (1%)  0 | 46 (58)  16 (20)  15 (19)  3 (4%)  0 | 97 (97)  3 (3)  0  0  0 | 71 (71)  15 (15)  10 (10)  4 (4%)  0 | 114 (96)  2 (2)  1(<1)  0  1 (<1) | 89 (76)  23 (19)  4 (3)  1 (<1)  1 (<1) | 93 (95)  3 (3)  0  0  2 (2) | 75 (77)  13 (13)  8 (8)  0  2 (2) |

**Supplementary Table 5.** Summary of ease of use questionnaire for each inhaler device; number (%) of asthma patients who responded to each question

|  |  | Substudy 1  (N=70) | | Substudy 2  (N=32) | | Substudy 3  (N=60) | |
| --- | --- | --- | --- | --- | --- | --- | --- |
|  |  | ELLIPTA | DISKUS | ELLIPTA | MDI | ELLIPTA | Turbuhaler |
| How do you rate ease of use of the inhaler? | Very easy/easy  Neutral  Difficult  Very difficult | 64 (92)  5 (7)  1 (1)  0 | 50 (71)  14 (20)  6 (9)  0 | 28 (88)  2 (6)  2 (6)  0 | 16 (50)  8 (25)  7 (22)  1 (3) | 58 (97)  1 (3)  0  0 | 37 (61)  16 (27)  6 (10)  1 (2) |
| How easily are you able to tell how many doses of medication are left in the inhaler? | Very easy/easy  Neutral  Difficult  Very difficult  Missing | 70 (100)  0  0  0 | 54 (77)  10 (14)  5 (7)  1 (1) | 32 (100)  0  0  0 | 26 (81)  5 (16)  1 (3)  0 | 60 (100)  0  0  0 | 22 (37)  21 (35)  16 (27)  1 (2) |
| How do you rate the ease of learning how to use the inhaler? | Very easy/easy  Neutral  Difficult  Very difficult | 68 (97)  2 (3)  0  0 | 57 (82)  12 (17)  1 (1)  0 | 32 (100)  0  0  0 | 21 (66)  7 (22)  4 (12)  0 | 58 (98)  1 (2)  0  0 | 37 (61)  14 (23)  9 (15)  0 |
| How do you rate the ease of handling the inhaler? | Very easy/easy  Neutral  Difficult  Very difficult | 67 (96)  3 (4)  0  0 | 52 (74)  13 (19)  5 (7)  0 | 31 (97)  1 (3)  0  0 | 19(60)  6 (19)  6 (19)  1 (3) | 59 (98)  1 (2)  0  0 | 41 (68)  13 (22)  5 (8)  1 (2) |
| How do you rate the ease of preparing the inhaler for use? | Very easy/easy  Neutral  Difficult  Very difficult | 66 (94)  0  4 (6)  0 | 57 (81)  11 (16)  2 (3)  0 | 31 (97)  1 (3)  0  0 | 27 (84)  3 (9)  2 (6)  0 | 59 (87)  7 (12)  1 (2)  0 | 41 (68)  15 (25)  4 (7)  0 |
| How do you rate the ease of holding the inhaler while using it? | Very easy/easy  Neutral  Difficult  Very difficult | 68 (97)  2 (3)  0  0 | 57 (81)  11 (16)  2 (3)  0 | 31 (97)  1 (3)  0  0 | 22 (69)  4 (13)  6 (19)  0 | 52 (87)  7 (12)  1 (2)  0 | 44 (73)  15 (25)  1 (2)  0 |
